# Interdisciplinary insights into public health challenges: A synthesis of research presented at the 2025 African Voices for Research Virtual Conference

**DOI:** 10.4102/jphia.v16i4.1506

**Published:** 2025-12-09

**Authors:** Qudus O. Lawal, Oluyemisi Olagunju, Dooshima D. Gbahabo, Obehi Osadolor, Henry O. Chukwudi, Elizabeth O. Omotola, Christiana T. Ekanade, Olabinri P. Folashade, Talatu R. Ndagi, Mary O. Adetula, Miracle C. Okeke, Ayobami O. Adeagbo, Rachael O. Oduyemi, Larry Ng’andu, Toluwalase S. Adekoya, Funmilayo G. Adebiyi, Esther O. Israel, Comfort O. Akanni, Morenike O. Folayan

**Affiliations:** 1 Department of Obstetrics and Gynaecology, Faculty of Clinical Sciences, Ambrose Ali University, Ekpoma, Nigeria; 2 Department of Nursing Science, Obafemi Awolowo University, Ile-Ife, Nigeria; 3 Department of Nursing Science, Faculty of Clinical Sciences, University of Lagos, Lagos State, Nigeria; 4 Department of Child Dental Health, University of Nigeria Teaching Hospital, Enugu, Nigeria; 5 Faculty of Dentistry, University of Port Harcourt, Port Harcourt, Nigeria; 6 Department of Chemical Sciences, Tai Solarin University of Education, Ijagun, Nigeria; 7 Department of Surveying and Geoinformatics, Faculty of Environmental Design and Management, Lead City University, Ibadan, Nigeria; 8 Department of Biochemistry, Membrane Biochemistry and Biophysics Research Laboratory, Ladoke Akintola University of Technology, Ogbomoso, Nigeria; 9 Department of Obstetrics and Gynaecology, Faculty of Clinical Sciences, Bayero University, Kano, Nigeria; 10 Department of Political Science, Obafemi Awolowo University, Ile-Ife, Nigeria; 11 Department of Internal Medicine, Faculty of Medicine, College of Medicine, Enugu State University of Science and Technology Teaching Hospital, Parklane, Nigeria; 12 Department of Nursing Science, National Institute of Health Research, Lagos, Nigeria; 13 Department of Community Health Nursing, Faculty of Nursing, Chrisland University, Abeokuta, Nigeria; 14 Department of Community Health Nursing, Faculty of Nursing, College of Medicine, University of Ibadan, Ibadan, Nigeria; 15 Laboratory Department, Faculty of Health Sciences, USAID - Action to HIV - Right to Care Zambia, Muchinga, Zambia; 16 Department of Psychology, Faculty of Social Sciences, Lagos State University, Ojo, Lagos, Nigeria; 17 Department of Animal Science, Faculty of Agriculture, University of Ibadan, Ibadan, Nigeria; 18 Centre for Foundation Education, Bells University of Technology, Ogun State, Nigeria; 19 Department of Medicine and Surgery, Faculty of Clinical Sciences, Olabisi Onabanjo University, Ago-Iwoye, Nigeria; 20 Department of Child Dental Health, Faculty of Dentistry, Obafemi Awolowo University, Ile-Ife, Nigeria

**Keywords:** gender equity, Universal Health Coverage (UHC), innovation, one health, environmental and occupational health, biomedical and nutritional research, Innovations in healthcare and technology, Social Determinant of Health (SDoH)

## Abstract

**Background:**

Public health challenges in Africa stem from a complex interplay of individual, environmental, socio-cultural, and health-system factors, requiring integrated, interdisciplinary approaches to enhance healthcare systems.

**Aim:**

This study synthesised evidence from interdisciplinary presentations at the 2025 African Voices for Research Virtual Conference to address public health challenges.

**Setting:**

The study was based on abstracts presented in sessions of the 2025 African Voices for Research Virtual Conference.

**Methods:**

An editorial narrative synthesis of all submitted abstracts was conducted. ChatGPT(OpenAI), supported the identification of patterns and themes, which were validated by the research team. Themes were grouped into broader categories reflecting major public health issues and potential solutions.

**Results:**

The study showed how healthcare access, education, environmental justice, gender equity and innovation intersect in advancing Universal Health Coverage and the Sustainable Development Goals. Using Social Determinants of Health and One Health perspectives, the findings showed how infrastructural gaps, financial barriers and limited health literacy drive unequal health outcomes. Case studies on maternal health and environmental exposures highlighted the influence of socio-economic inequality and structural violence on population well-being. Gender disparities were prominent, especially among rural women facing political exclusion, violence and restricted access to care. Innovation in healthcare delivery, especially AI-enabled tools and diagnostic platforms such as GeneXpert, was recognised for its potential to strengthen health-system resilience.

**Conclusion:**

This study offers an overview of emerging research themes from the conference and provides insight into future research priorities.

**Contribution:**

This research highlights how healthcare access, environmental and gender inequities, and technological innovation shape population health across Africa.

## Introduction

Public health research plays a pivotal role in identifying, understanding and addressing the multifaceted challenges that impact communities, particularly in low-resource settings where health inequities are most pronounced.^1^ It provides the empirical foundation needed to identify pressing health issues, uncover the social, environmental and economic determinants driving them, and design evidence-based interventions that are contextually relevant and culturally appropriate. The evidence generated also enables policymakers and practitioners to allocate scarce resources efficiently, prioritise high-impact strategies and monitor progress over time.^2,3,4,5^ In addition, it empowers communities by generating locally driven solutions, fostering accountability, and informing policy reforms that promote health equity and resilience.^6,7,8^ By bridging the gap between knowledge and action, public health research not only reveals what needs to change, but it also illuminates how to make that change possible.^9^

At the 2025 African Voices for Research Virtual Conference, the 17 abstracts represent a broad yet interconnected investigation into pressing health and social challenges across various Nigerian communities and, in one case, Zambia. Although the individual studies span diverse disciplines – from oral health and environmental safety to gender equity, maternal care and digital innovation in diagnostics – they collectively aim to generate context-specific evidence that can inform policy, improve service delivery and ultimately enhance population health outcomes in low-resource settings. They collectively emphasise the intersection of socio-economic, cultural and systemic factors that shape health outcomes in these regions. The compiled research employs various methodologies, including cross-sectional surveys, quasi-experimental designs, phenomenological inquiries and machine learning models, to explore topics such as barriers to maternal care, occupational hazards among e-waste workers, antimicrobial resistance and the psychosocial impacts of intimate partner violence. The presentations highlight the need for an interdisciplinary approach to public health response.

This editorial of the abstracts (Online Appendix 1) presented at the 2025 African Voices for Research Virtual Conference aims to explore and address the determinants of health and well-being in vulnerable populations through empirical inquiry and applied research. We conducted an inductive analysis of the abstracts. This method is grounded in the principle that themes and insights should emerge organically from the data rather than being imposed by the researcher in advance. Through repeated engagement with the data presented and engagement with the abstract presenters, we identified significant textual patterns.

The abstracts from the interdisciplinary presentations were fed into ChatGPT to support the synthesis of key points.^10^ The model first generated a set of preliminary themes from the abstracts. With the input and guidance of the 19 members of the research team, these themes were refined and organised into broader thematic categories. The team then systematically reviewed the Artificial Intelligence (AI)-generated outputs, cross-validated them against the raw abstracts, and refined them further through consensus discussions. The themes were consolidated into five overarching public health themes.

## Emerging themes

**FIGURE 1:**
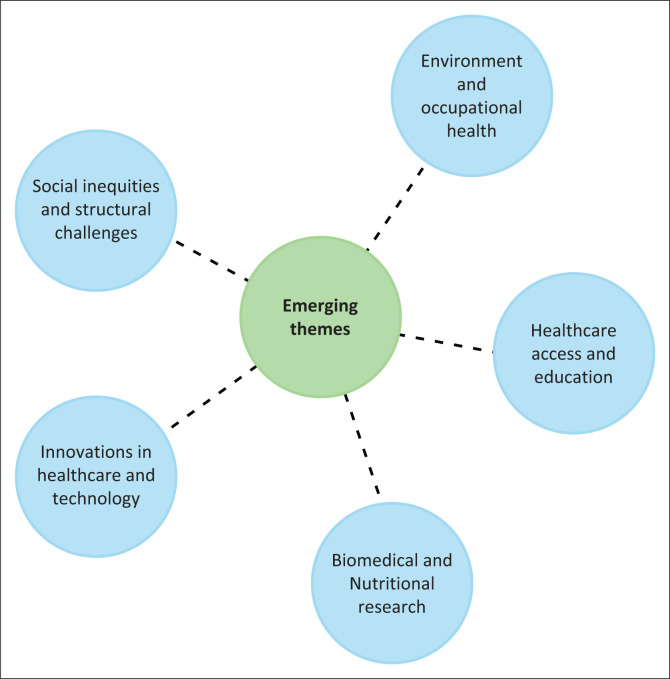
Emerging themes from the conference abstracts clustered into five overarching dimensions of public health (image generated by the research team using ChatGPT [OpenAI, GPT-5]).

The following themes emerged from the analysis of conference abstracts (Figure 1): (1) healthcare access and education, (2) environmental and occupational health, (3) social inequities and structural challenges, (4) innovations in healthcare and technology, and (5) biomedical and nutritional research.

### Healthcare access and education

Healthcare access and education are inextricably linked pillars of public health, particularly in resource-limited settings such as Nigeria. This connection is clearly illustrated across the 17 abstracts examined, which together provide compelling evidence that these two domains operate synergistically to address health disparities, empower communities and support the development of sustainable health systems. A structured discussion of the insights drawn from these studies reveals key challenges, identifies promising strategies and suggests important future directions for public health interventions.

Education serves as a critical determinant of health access by enhancing health literacy, enabling individuals to recognise symptoms, seek care promptly and adhere to preventive measures.^11^ For example, Chidiebere et al. (Online Appendix 1, Abstract 009) found that women in rural areas who had attained higher levels of education exhibited significantly better knowledge of pulmonary tuberculosis (PTB) and more consistent access to primary healthcare services. Similarly, Oduyemi et al. (Online Appendix 1, Abstract 012) suggested that peer education programmes were associated with improved awareness of breast cancer and the practice of self-examination among rural women, an approach that effectively bridged the gap created by limited formal healthcare access.

Chukwudi and Osagbemiro (Online Appendix 1, Abstract 002) revealed low awareness of the risks associated with tetracycline use among pregnant women, while Lawal et al. (Online Appendix 1, Abstract 006) pointed to limited ethics training for medical and dental students, gaps that reflect broader systemic failings in health education. Misinformation was also a key concern, as seen in the abstract by Ekanade and Adenekan (Online Appendix 1, Abstract 004), where e-waste workers in Lagos displayed varying degrees of risk awareness. Despite experiencing high rates of respiratory and dermatological issues, 56.3% of respondents were unconcerned about the health implications of their work, illustrating the dire need for targeted educational outreach.

Conversely, healthcare access itself can act as a catalyst for health literacy and social development.^12,13^ Akanni and Adetoye (Online Appendix 1, Abstract 017) highlighted how children living with sickle cell disease often experience developmental delays because of inadequate access to diagnostic and therapeutic services, ultimately affecting their ability to participate in educational activities. Psychological trauma, such as that resulting from intimate partner violence, was found to be similarly associated with detrimental effects on victims’ education and social outcomes, as seen in Israel (Online Appendix 1, Abstract 016). These findings underscore the cyclical relationship between health and education, where deficits in one can profoundly disrupt the other.

Despite the critical importance of healthcare and health literacy, structural and social barriers that hinder healthcare access and exacerbate educational gaps remain.^14^ Infrastructural deficits were evident in Yobe State, where health facilities were plagued by shortages of essential drugs, non-functional equipment and an absence of emergency blood transfusion services, as reported in Ndagi et al. (Online Appendix 1, Abstract 007). These challenges directly undermined the delivery of maternal and child health services. Geographic and financial constraints compounded the problem,^15,16^ especially in rural areas, as observed in Enugu (Osadolor and Osadolor, Online Appendix 1, Abstract 001) and Imo states (Chidiebere et al., Online Appendix 1, Abstract 009), where communities faced prohibitive transportation costs and income disparities that limited their ability to access healthcare.

Cultural norms and societal expectations also posed significant obstacles.^17^ In Osun State, patriarchal norms were shown to restrict women’s participation in politics (Adetula, Online Appendix 1, Abstract 008), reflecting broader issues of gender inequity that influence women’s autonomy in making healthcare decisions. Adeagbo et al. (Online Appendix 1, Abstract 011) further illustrated how cultural restrictions were a major factor in non-compliance with postnatal care, alongside logistical concerns such as transportation and work obligations.

In response to these complex challenges, integrated public health strategies are essential. Community-based education programmes, particularly those led by peers, have demonstrated notable success.^18,19^ The breast cancer intervention in Ogun State (Oduyemi et al., Online Appendix 1, Abstract 012) exemplifies the power of training local educators to disseminate critical health information. This model could be adapted for use in other health domains, such as the prevention of dental caries (Osadolor and Osadolor, Online Appendix 1, Abstract 001) or increasing awareness about tetracycline misuse (Chukwudi and Osagbemiro, Online Appendix 1, Abstract 002).

Integrating health education into schools and workplaces is another promising avenue.^18,20^ Lawal et al. (Online Appendix 1, Abstract 006) underscored the value of embedding structured ethics training within undergraduate medical curricula, while Ekanade and Adenekan (Online Appendix 1, Abstract 004) highlighted the need for improved occupational health education among e-waste workers. The examples from these preliminary studies point to the importance of tailoring education to specific settings and populations. Immersive technologies, such as virtual reality, could be used to enhance training, particularly in areas such as trauma care.^21^

Strengthening healthcare systems also requires targeted workforce training and strategic deployment of resources.^22^ As observed by Ogbu et al. (Online Appendix 1, Abstract 010), improving the competence of nurses in managing rape survivor assessments may be associated with enhanced care quality, while Ng’andu et al. (Online Appendix 1, Abstract 013) emphasised the importance of training health workers to effectively use diagnostic tools such as GeneXpert for tuberculosis in Zambia. Technology-driven solutions offer further potential; for instance, the DevSickleNet AI model (Akanni and Adetoye, Online Appendix 1, Abstract 017) shows promise in democratising access to early diagnostics in underserved areas.

Policy-level changes and cross-sectoral collaboration are equally vital.^22^ Adetula (Online Appendix 1, Abstract 008) called for gender-sensitive policies to support women’s leadership and health access, while Ndagi et al. (Online Appendix 1, Abstract 007) emphasised the need for political commitment to maternal health. Public-private partnerships, such as collaborations with non-governmental organisations, could help address financial barriers (Online Appendix 1, Abstract 011) or support environmental health interventions, including pesticide clean-up (Omotola, Online Appendix 1, Abstract 003).

Looking ahead, future public health interventions must be context-specific, adaptable and equity-driven.^23^ This would require that rural-urban disparities and cultural resistance be addressed through culturally appropriate, context-specific interventions. For example, mobile clinics could bring care closer to remote communities in Enugu state (Osadolor and Osadolor, Online Appendix 1, Abstract 001), and telehealth platforms could be used to extend ethics training to medical students in distant regions (Lawal et al., Online Appendix 1, Abstract 006). Mobile health (mHealth) tools such as SMS campaigns and AI-powered chatbots could serve as scalable platforms to disseminate accurate health information and provide real-time support, particularly to marginalised and vulnerable populations.

For many countries in Africa, however, sustainable funding remains a pressing public health concern and a limiting factor for progressive health responses.^24^ Persistent shortages of diagnostic kits in Zambia (Ng’andu et al., Online Appendix 1, Abstract 013) and essential medications in Yobe State, Nigeria (Ndagi et al., Online Appendix 1, Abstract 007) underscore the consequences of chronic underinvestment. The studies suggest that while prioritisation of domestic health financing and international development aid can help bridge these resource gaps, research and innovation are important. Research can help identify low-cost solutions tailored to context-specific needs. For example, while small scale research on the use of AI technologies such as DevSickleNet offer exciting prospects for the early diagnosis of sickle cell disease (Akanni and Adetoye, Online Appendix 1, Abstract 017), and other initiatives like peer education (Oduyemi et al., Online Appendix 1, Abstract 012) and ethics curricula (Lawal et al., Online Appendix 1, Abstract 006) can have impact on health outcomes and professional behaviour, long-term studies are necessary to evaluate the enduring impact of these programmes and how to scale them up for national impact.

Healthcare access and education must be viewed not as separate silos but as mutually reinforcing components of a comprehensive public health strategy. Effective responses require a commitment to integration – embedding health education across community, school and workplace environments; to equity – addressing the needs of marginalised populations, including rural women, informal workers and survivors of violence. In addition, there is a need for innovations, leveraging technology and data to close systemic gaps. The collective findings from these abstracts affirm that progress depends on multisectoral collaboration, sustained financial investment, and a deep commitment to dismantling the structural and educational barriers that hinder health equity to build resilient and inclusive health systems for the future.

### Environmental and occupational health

The conference abstracts underscore pressing environmental and occupational health challenges that disproportionately affect low- and middle-income countries. These studies collectively expose gaps in policy enforcement, infrastructure and public awareness, while also pointing to opportunities for strategic interventions.

With respect to environmental health, Omotola (Online Appendix 1, Abstract 003) presents compelling evidence of ongoing contamination from organochlorine pesticides in surface sediments across Ijebu-Ode, Ogun State, Nigeria. Despite global bans on substances such as β-BHC, the study detected concentrations as high as 28.13 mg/kg, revealing persistent environmental pollution. These organochlorine pesticides, classified as persistent organic pollutants, can bioaccumulate in ecosystems, ultimately entering human populations through water, soil and food chains. The absence of significant variations across sampling sites suggests that the pollution is widespread, likely driven by illegal pesticide use, inadequate disposal systems or legacy agricultural runoff. The implications are alarming: chronic exposure to organochlorine pesticides is linked to serious health outcomes, including cancers, endocrine disruption and neurotoxicity. The findings also point to policy gaps – specifically, weak enforcement mechanisms and poor monitoring systems. Considering this, the study calls for immediate remediation efforts, such as the adoption of bioremediation technologies and the strengthening of regulatory frameworks governing pesticide use and waste management. It also recommends launching community awareness campaigns to promote safer agricultural practices and investing in environmental surveillance systems to identify and respond to pollution hotspots.

On occupational health, Ekanade and Adenekan (Online Appendix 1, Abstract 004) investigate the health risks faced by e-waste workers in Lagos State. The study paints a grim picture of workplace conditions, with 54% of respondents reporting physical symptoms such as skin and eye irritation, respiratory issues and body pain. Equally concerning is the widespread lack of awareness and protective measures: 56% of workers expressed indifference towards the health implications of their jobs, and 63.5% admitted to not using appropriate safeguards, such as changing out of contaminated clothing. A significant correlation (r = 0.755, p < 0.001) was found between occupational exposures and reported health problems, underscoring the systemic neglect of worker safety in this informal sector. These findings reflect broader trends observed in other Global South e-waste hubs such as South Asia (India, Pakistan, Bangladesh, Sri Lanka) and sub-Saharan African countries (Ghana, Nigeria, South Africa),^25,26,27,28^ where economic pressures often force workers to prioritise income over health.^29^ The study advocates for the implementation of comprehensive workplace safety protocols, including the provision of personal protective equipment and improved ventilation. It also emphasises the importance of partnerships with non-governmental organisations to provide regular health screenings and training in hazard mitigation and calls for formalising the e-waste recycling sector to ensure regulatory oversight and protect vulnerable workers.

Ng’andu et al. (Online Appendix 1, Abstract 013) shift the focus to the intersection of infrastructure, diagnostics and healthcare equity. In this study, researchers examined the utilisation of GeneXpert machines in Zambian hospitals and uncovered stark disparities. While the urban hospital in Isoka operated at 166% of its diagnostic capacity, rural Muyombe struggled to reach 71%, hindered by persistent power outages and stockouts of essential reagents. These operational inequities exemplify the environmental justice challenges facing rural communities, where inadequate infrastructure limits access to timely disease diagnostics for conditions like tuberculosis and human immunodeficiency virus (HIV). Healthcare workers in these settings are burdened by burnout and diminished capacity to respond to health threats, further widening the gap in service delivery. The study highlights the need for investments in renewable energy sources, such as solar power, to stabilise electricity in rural facilities. It also calls for strengthening supply chains through public–private partnerships to ensure the availability of diagnostic materials and support consistent testing services.

Several cross-cutting themes emerge from the findings of the studies presented. Weak policy implementation remains a critical issue, as ineffective enforcement of environmental and occupational regulations continues to expose populations to avoidable risks.^30^ At the same time, abstracts such as that by Oduyemi et al. (Online Appendix 1, Abstract 012) on the impact of peer-led breast cancer training demonstrate the power of community engagement and grassroots education in catalysing behavioural change. Ultimately, these challenges demand a coordinated, multisectoral approach involving governments, civil society, academic institutions and private sector stakeholders.

The evidence from these abstracts points to a clear and urgent need for action to address the environmental and occupational health disparities in settings with limited resources. While these studies highlight localised issues, they also reflect systemic inequalities in global health governance.^31^ Scalable and context-appropriate solutions are needed to address the health disparities in Africa. These should be complemented with long-term health impact assessments and robust policy advocacy to ensure sustainable improvements. Remediation, worker protection and infrastructure development must be central to any effort aiming to promote equity and health in the face of these enduring challenges. In addition, policymakers need to integrate gender-sensitive approaches into health strategies and to strengthen regulations on pesticide use and waste management.

### Social inequities and structural challenges

The conference programme weaves a powerful narrative about the complex interplay between social inequities and structural challenges, revealing how systemic neglect and entrenched norms continue to marginalise vulnerable populations. These challenges cut across critical sectors such as healthcare, education, environmental management, gender equity and governance, reinforcing cycles of disadvantage.

Disease surveillance and diagnosis are hindered by unreliable power supply and stockouts that disrupt the use of GeneXpert machines for tuberculosis testing (Ng’andu et al., Online Appendix 1, Abstract 013). Even where health services are technically available, access remains elusive. In Ibadan, women’s non-compliance with postnatal care appointments has been attributed to transportation difficulties, cultural restrictions and work responsibilities – clear indicators of broader socio-economic inequities (Adeagbo et al., Online Appendix 1, Abstract 011). These studies highlighted structural deficits in the delivery of healthcare in Africa: chronic underinvestment in healthcare systems, including infrastructure and workforce development, continues to sustain deep health disparities, particularly for women, children and rural populations.

Gender inequities permeate nearly every domain explored in the conference. In Osun State, Nigeria, women’s political representation remains alarmingly low: they hold just 15.9% of appointed positions and none of the elected offices, a reflection of entrenched patriarchal systems and financial constraints that exclude women from governance (Adetula, Online Appendix 1, Abstract 008). The repercussions of gender-based violence also resonate powerfully. In Lagos State, Nigeria, children exposed to intimate partner violence face long-term academic and psychological harm, underscoring how gendered abuse perpetuates intergenerational trauma and disadvantage (Israel, Online Appendix 1, Abstract 016). Gender dynamics also shape occupational risks. At Lagos’s Ladipo Market, a male-dominated e-waste sector exposes workers to serious health risks because of lax safety regulations, with many labourers underestimating the dangers despite evident respiratory and skin conditions (Ekanade and Adenekan, Online Appendix 1, Abstract 004). These realities call for urgent reform: deeply rooted gender norms, insufficient legal protections and economic disempowerment continue to sustain gender-based disparities, demanding both policy change and grassroots mobilisation.

Socio-economic determinants such as income, education and housing consistently emerged as central to health and opportunity. In Orlu, Imo State, Nigeria, poor awareness of PTB and limited healthcare access were strongly associated with low-income and educational levels, revealing how poverty stifles health-seeking behaviour and deepens vulnerability (Chidiebere et al., Online Appendix 1, Abstract 009). In Lagos, the long-term consequences of socio-economic deprivation were illustrated in studies linking childhood emotional neglect to coercive and violent romantic relationships in adulthood (Adekoya et al., Online Appendix 1, Abstract 014). These examples underscore the cumulative, life-course impact of poverty and inequality, and the necessity for integrated responses that address housing, employment and education alongside health.

Technological and resource gaps amplify these challenges. In settings where traditional healthcare resources are limited, innovations such as DevSickleNet – an AI model for sickle cell disease screening – highlight the promise of technology in bridging diagnostic divides (Akanni and Adetoye, Online Appendix 1, Abstract 017). However, such solutions remain largely inaccessible in low-resource areas, pointing to a digital divide that reinforces existing inequities.^32^ Meanwhile, the turn towards polyherbal medicine formulations (Olabinri et al., Online Appendix 1, Abstract 005) reflects deeper structural and cultural dynamics shaping healthcare choices in African communities. On the one hand, this shift underscores long-standing cultural preferences for traditional medicine, which is often viewed as more accessible, spiritually attuned and embedded in local knowledge systems.^33^ Polyherbal formulations, in particular, are rooted in indigenous pharmacological practices where the synergistic effect of multiple herbs is believed to enhance therapeutic efficacy and reduce side effects.^34^ On the other hand, the growing reliance on such alternatives also signals critical systemic gaps in the availability, affordability and acceptability of conventional treatments in many low-resource settings where the health systems remain underfunded, and essential medicines may be unavailable or unaffordable.^35,36^

To address these deeply rooted structural challenges, findings from the conference suggest several equity-driven solutions. Multisectoral collaboration is essential by integrating healthcare, education and environmental policies to tackle root causes. For instance, combining antenatal care services with drug safety education could improve both maternal and child health outcomes. Policy enforcement must be strengthened, particularly around environmental protections, labour conditions and gender quotas in leadership. Infrastructure investment is also critical, particularly in rural healthcare systems, diagnostic technology powered by renewable energy, and improved transportation networks to facilitate care access. Finally, there is a need for rigorous scientific validation of polyherbal formulations to ensure their safety, efficacy and quality. At the same time, integrative healthcare models that recognise the legitimacy of traditional medicine, alongside biomedical care, could offer more culturally resonant and accessible solutions. A key message from this research is that structural failings, whether in governance, resource allocation or cultural practices, drive social inequities in profound and intersecting ways. Tackling these issues requires not only reform at the institutional level but also sustained engagement with communities and a commitment to justice-oriented policy.

### Innovations in healthcare and technology

The conference presentations illuminate a transformative era in healthcare, where technological innovation is becoming central to addressing complex health challenges in resource-limited contexts. The presentations highlight advancements in diagnostic methods, computational modelling and telehealth. These developments are not isolated; rather, they demonstrate an interdisciplinary approach aimed at improving health systems through evidence-based, context-sensitive and scalable innovations.

One of the most significant themes is the advancement in diagnostic technologies and computational methods. Omotola (Online Appendix 1, Abstract 003) and Olabinri et al. (Online Appendix 1, Abstract 005) demonstrate how gas chromatography-mass spectrometry is being used to detect organochlorine pesticide residues in sediments and to identify bioactive compounds in herbal formulations. These analytical methods are critical tools for environmental monitoring and drug discovery, particularly in areas battling pollution and the rising tide of antimicrobial resistance. Meanwhile, Ng’andu et al. (Online Appendix 1, Abstract 013) focus on the implementation of the GeneXpert platform in rural Zambia for tuberculosis and viral load testing. While the technology holds promise for improving diagnostic access in underserved regions, its impact is hindered by infrastructure challenges, such as power instability and supply chain bottlenecks. These limitations reinforce the necessity for sustainable solutions such as solar-powered diagnostic tools^37^ and decentralised procurement systems.^38^

The integration of Artificial Intelligence (AI) and predictive modelling into healthcare is also gaining momentum. Akanni and Adetoye (Online Appendix 1, Abstract 017) introduce DevSickleNet, an AI model with an 85.4% accuracy rate in predicting developmental delays in children with sickle cell disease. By synthesising clinical data, caregiver inputs and developmental milestones, the model enables early intervention and underscores the potential of AI in personalised care. In addition, Olabinri et al. (Online Appendix 1, Abstract 005) illustrate the application of molecular docking in silico to identify herbal compounds with antimicrobial properties, specifically highlighting 4-cholesten-3-one semicarbazone as a potential deoxyribonucleic acid (DNA) gyrase inhibitor. These computational approaches streamline the drug discovery process and offer cost-effective alternatives to traditional lab-based experiments.^39^

When it comes to telehealth and mobile health solutions, the potential to bridge gaps in access is evident. Ndagi et al. (Online Appendix 1, Abstract 007), Chidiebere et al. (Online Appendix 1, Abstract 009) and Adeagbo et al. (Online Appendix 1, Abstract 011) explore the systemic challenges facing maternal care, tuberculosis knowledge and postnatal follow-ups, respectively. Telemedicine platforms and mobile clinics equipped with portable diagnostic tools like GeneXpert can mitigate some of these barriers.^40,41^ Moreover, AI-driven mobile applications could support triaging of high-risk pregnancies and TB cases,^42,43^ offering a lifeline to communities otherwise excluded from conventional healthcare systems. It should be noted, however, that these observations are forward-looking opinions derived from the abstracts rather than empirically tested study outcomes.

Yet, these innovations are not without challenges. Infrastructural deficits such as inconsistent electricity and poor internet connectivity limit the reach of tools such as GeneXpert and telehealth platforms (Ng’andu et al., Online Appendix 1, Abstract 013; Akanni and Adetoye, Online Appendix 1, Abstract 017). Ethical and privacy concerns surrounding the use of AI and digital data require robust governance frameworks to protect patient confidentiality.^44^ Furthermore, cultural and socio-economic factors that limit health literacy (Adetula, Online Appendix 1, Abstract 008) and entrenched gender inequities (Adeagbo et al., Online Appendix 1, Abstract 011) call for interventions that are grounded in local realities and led by communities themselves.

Looking ahead, several future directions can be envisaged from the conference findings. Hybrid diagnostic models that combine AI with human oversight can enhance both accuracy and trust in digital tools. The development of sustainable technologies as solar-powered medical devices and biodegradable environmental sensors will be crucial for long-term impact on health. Finally, the use of synthetic data and open-source platforms could democratise access to advanced tools and improve healthcare delivery in low-income settings.^45^

### Biomedical and nutritional research

The conference programme brought to light pivotal developments and persistent challenges in biomedical and nutritional research, emphasising the importance of interdisciplinary approaches to improving public health and promoting sustainable agricultural practices. This synthesis offers a narrative exploration of the central themes, their implications and future directions.

In the realm of biomedical research, issues related to public health and broader societal challenges were especially prominent. Oral health and medication safety were focal points. A study on dental caries in rural Nigerian adolescents (Osadolor and Osadolor, Online Appendix 1, Abstract 001) revealed a notably low prevalence of dental caries of 8.4%, deviating from global trends. This unexpected outcome suggests potential protective factors specific to the local context, such as dietary patterns. Interestingly, the study found no significant correlation between dental caries and socio-economic status or oral hygiene practices, pointing to the need for longitudinal research to further investigate possible environmental or genetic contributors. On the other hand, Chukwudi and Osagbemiro (Online Appendix 1, Abstract 002) exposed significant gaps in antenatal education, underscoring the urgent need to incorporate medication safety education into antenatal services to reduce the risks associated with self-medication and improve maternal-child health outcomes.

The study on e-waste workers (Ekanade and Adenekan, Online Appendix 1, Abstract 004) documented high incidences of respiratory and skin disorders, linked with a strong correlation (r = 0.755) between exposure and reported symptoms. The findings highlight an urgent need for improved protective equipment and regulatory oversight in informal sectors. Similarly, the detection of banned organochlorine pesticides in Nigerian aquatic sediments (Omotola, Online Appendix 1, Abstract 003) signals dangerous lapses in environmental management, highlighting the need for robust remediation strategies such as bioremediation and tighter regulatory enforcement to curb contamination of the food chain. Maternal and child health can also be improved through the development of a comprehensive Reproductive Maternal Child Health roadmap informed by community engagement (Ndagi et al., Online Appendix 1, Abstract 007), and the deployment of community health workers to address barriers to postnatal care (Adeagbo et al., Online Appendix 1, Abstract 011). Finally, school-based emotional support programmes to interrupt intergenerational cycles of trauma are also needed (Adekoya et al., Online Appendix 1, Abstract 014).

On the nutritional front, innovations in animal husbandry were explored. Adebiyi et al. (Online Appendix 1, Abstract 015) examined the effects of lime juice supplementation in swine diets, revealing improved immune biomarkers (monocytes, neutrophils) and increased globulin levels without adverse effects. These findings support the growing shift towards natural feed additives as a means of enhancing livestock health^46,47^ and reducing reliance on antibiotics.^48^ Future research is needed to fine-tune dosage and assess long-term impacts on animal growth and productivity.

## Discussion

A prominent theme emerging from the 2025 African Voices for Research Virtual Conference is the complex relationship between healthcare access, education and the pursuit of Universal Health Coverage. The Social Determinants of Health (SDOH) framework^49^ provides a useful lens to understand how infrastructural deficits, financial barriers and limited health literacy, as illustrated in studies on maternal care and medication safety, shape uneven health outcomes. These issues are compounded by socio-economic inequalities and cultural factors that influence health-seeking behaviours and perceptions of care.^50^ The study findings point to a need for culturally tailored health education initiatives that inform and empower individuals to make informed health decisions. This should be complemented with interventions such as artificial intelligence, mobile clinics, telemedicine platforms and community-based health education programmes that bridge access gaps, particularly in underserved and rural areas.^51^ These solutions, when combined with systemic investments in workforce capacity and health infrastructure development, can directly reverse the stagnation of UHC goals by ensuring that essential health services are accessible, affordable and acceptable to all, regardless of geographic or socio-economic status.^22,52^

Another highlight of the article is the recognition of the interconnectedness of human, animal and environmental well-being as exemplified by findings on organochlorine pesticide residues (Omotola, Online Appendix 1, Abstract 003), and ecosystems and hazardous exposures among e-waste workers (Ekanade and Adenekan, Online Appendix 1, Abstract 004). The study on e-waste workers also reflects the structural violence endured by informal labourers, where economic disenfranchisement translates into disproportionate exposure to environmental harms.^53^ In this system, invisible, normalised structural harms, like poverty and hunger, are linked to more overt and visible harm, like the health problems reported in the study.^54^ The evolving concept of the One Health approach^55^ increasingly transcends its foundational focus on biological etiological factors, embracing a more holistic understanding that integrates structural and socio-economic determinants of health. These two studies exemplify the expanded lens by underscoring how systemic inequities and structural violence (economic disenfranchisement, informal labour exploitation and regulatory neglect) amplify health risks across human, animal and environmental spheres.

Thus, structural inequalities mediate environmental harm. Organochlorine pesticides, although banned or restricted in many regions, persist in ecosystems because of lax regulations, corporate prioritisation of agricultural productivity over safety and economic pressures that push small-scale farmers towards cheaper, hazardous chemicals. Similarly, e-waste workers, often marginalised informal labourers, endure toxic exposures because systemic poverty and a lack of alternative livelihoods force them into unsafe recycling practices. Both scenarios highlight a cycle where structural issues, such as neoliberal economic policies, weak governance and globalised waste and/or chemical trade, disproportionately burden vulnerable populations while degrading ecosystems. In addition, these cases illustrate how invisible structural harms (poverty, hunger, labour exploitation) may manifest as visible health crises (pesticide-linked cancers, e-waste-induced neurotoxicity).

Thus, the One Health approach, when applied through a structural justice lens, challenges the fragmentation of human, animal and environmental health agendas. It demands interventions that address root causes, such as equitable resource distribution, strengthened labour rights and transnational accountability for pollution. By bridging ecological and social determinants, One Health can evolve into a framework that not only diagnoses interconnected risks but also dismantles the systems perpetuating them. This call for the reimagining of One Health as a tool for transformative equity, one that engages the community, ensuring solutions to zoonotic threats or chemical contamination are inseparable from efforts to rectify the power imbalances and economic structures that normalise harm.^56^

Social inequities that require innovative solutions, especially gender-based, were another highlight of the conference. Drawing from feminist theory^57^ and intersectionality,^58^ several studies exposed how entrenched patriarchal norms and socio-economic marginalisation intersect to compound disadvantage for women, particularly those in rural areas. The research presented on political exclusion (Adetula, Online Appendix 1, Abstract 008), intimate partner violence (Israel, Online Appendix 1, Abstract 016) and maternal health barriers (Ndagi et al., Online Appendix 1, Abstract 007) calls for gender-sensitive policymaking and psychosocial support systems that advance the goals of Sustainable Development Goal 5 on gender equality. These findings also echo the African Centres for Disease Control’s New Public Health Order, emphasising community-led and equity-focused responses to public health challenges.^59^ Innovations such as peer education and AI tools such as DevSickleNet signal (Akanni and Adetoye, Online Appendix 1, Abstract 017) a commitment to harnessing local agency for sustainable health gains.

The role of innovation in healthcare delivery was particularly notable. While studies acknowledged infrastructural constraints to deploying technologies such as AI diagnostics (Akanni and Adetoye, Online Appendix 1, Abstract 017) and GeneXpert (Ng’andu et al., Online Appendix 1, Abstract 013), they also highlighted these tools’ transformative potential. These technologies enhance individual capabilities to achieve better health outcomes and contribute to the resilience of health systems even in remote and underserved areas, especially in the face of threats such as tuberculosis and sickle cell disease.^51^ Such innovations speak directly to SDG 3 and SDG 9, as well as the Africa CDC’s vision of a health-secure Africa through strengthened diagnostics and decentralised care.^60^

Herbal medicinal product use has increased over the years because of their low toxicity, ease of access and potential effect on multidrug-resistant pathogens.^61,62,63^ As a result of the synergistic effects of polyherbal formulations that contribute to the drug’s efficacy, herbal medicine research is highly recommended.^64,65^ Thus, the identification of metabolites present in natural products is of utmost importance because it drives drug development processes.^66,67^ The findings of the research presented by Olabinri et al. (Online Appendix 1, Abstract 05) revealed that the polyherbal formulation has compounds capable of causing an enhanced antibacterial effect than the tested antibiotics, and this can be evaluated in further scientific studies.

### Limitations

This editorial synthesis is based on conference abstracts, with varying methodologies. As such, the findings should be interpreted with caution because of potential heterogeneity and possible selection bias in the submitted abstracts. Artificial intelligence tools were used to assist in organising and synthesising the material; however, all outputs were verified by human reviewers to ensure accuracy and contextual validity.

## Conclusion

The conference findings suggest an urgent need for multisectoral coordination, where health policies must intersect with education, environmental management and gender equity strategies. Realising the ambitions of the Sustainable Development Goals and the Africa CDC’s New Public Health Order will require bold steps in policy integration, community empowerment and technological inclusivity. Grounding interventions in frameworks such as SDOH, Planetary Health and the Capability Approach offers a roadmap for African nations to navigate contemporary health challenges, ranging from climate change to antimicrobial resistance, while advancing equity and sustainable development. Moving forward, research must prioritise scalability, sustainability and equity to ensure that innovations translate into measurable improvements in population health.
